# Reversible binding of divalent cations to *Ductin* protein assemblies—A putative new regulatory mechanism of membrane traffic processes

**DOI:** 10.3389/fmolb.2023.1195010

**Published:** 2023-05-09

**Authors:** Krisztina Sebők-Nagy, András Blastyák, Gábor Juhász, Tibor Páli

**Affiliations:** ^1^ Institute of Biophysics, Biological Research Centre, Eötvös Loránd Research Network, Szeged, Hungary; ^2^ Institute of Genetics, Biological Research Centre, Eötvös Loránd Research Network, Szeged, Hungary

**Keywords:** *Ductin*, V-ATPase, F-ATPase, ATP synthase, gap-junction, autophagy, mitochondrial permeability transition pore, divalent cation

## Abstract

*Ductins* are a family of homologous and structurally similar membrane proteins with 2 or 4 trans-membrane alpha-helices. The active forms of the *Ductins* are membranous ring- or star-shaped oligomeric assemblies and they provide various pore, channel, gap-junction functions, assist in membrane fusion processes and also serve as the rotor c-ring domain of V-and F-ATPases. All functions of the *Ductins* have been reported to be sensitive to the presence of certain divalent metal cations (Me^2+^), most frequently Cu^2+^ or Ca^2+^ ions, for most of the better known members of the family, and the mechanism of this effect is not yet known. Given that we have earlier found a prominent Me^2+^ binding site in a well-characterised *Ductin* protein, we hypothesise that certain divalent cations can structurally modulate the various functions of *Ductin* assemblies via affecting their stability by reversible non-covalent binding to them. A fine control of the stability of the assembly ranging from separated monomers through a loosely/weakly to tightly/strongly assembled ring might render precise regulation of *Ductin* functions possible. The putative role of direct binding of Me^2+^ to the c-ring subunit of active ATP hydrolase in autophagy and the mechanism of Ca^2+^-dependent formation of the mitochondrial permeability transition pore are also discussed.

## *Ductins* in membrane-transport processes

**FIGURE 1:**
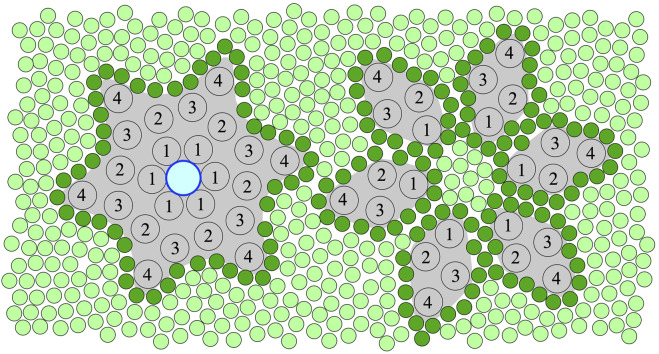
An illustration of the top view of the hexameric ring assembly (left) and separated monomers (right) of 16-kDa, 4 trans-membrane alpha-helix (TMH) Ductin proteins in a membrane. The central pore, which can only form in the ring assembly, is shown with an outlined blue circle (left). The numbers indicate the TMHs of the monomers in the sequential order. Lipids are shown as green circles, and the first shell or annular lipids contacting the protein surface are shown in darker green. According to the hypothesis of this paper, binding of specific divalent cations shifts the equilibrium towards the stable hexameric ring assembly (left) needed for both the pore, gap-junction and rotor functions of these Ductin proteins. However, inter- and intra-membrane rearrangement, which is needed for putative membrane fusion-related functions, is only possible in a loosely assembled or fully disassembled state, assuming low concentration of the divalent cations.

In the ‘80s a 16-kDa protein was purified from the presynaptic plasma membranes of the electric organ of *Torpedo marmorata*. The protein was called “mediatophore” because it was shown to mediate membrane translocation of acetylcholine (ACh) in a calcium-dependent manner. The active form of the mediatophore was an oligomeric ring, not linked by disulphide bonds but it required certain native lipids for function (Israël et al., 1986; Israël et al., 1988). It showed high sequence homology with the bovine chromaffin granule protonophore and subunits of the proteolipid c-ring of the yeast vacuolar proton-ATPase (V-ATPase) (Birman et al., 1990). It was also demonstrated that calcium-induced occurrence of intramembranous particles was conditional to ACh release from proteoliposomes equipped with mediatophore (Brochier et al., 1992). Although N,N’-dicyclohexylcarbodiimide (DCCD, a proton-translocation blocker of V-ATPase, which targets carboxyl groups within the membrane) was shown to bind to mediatophore, it still had the ability of calcium-dependent ACh translocation, suggesting that different protein domains were involved in ACh and proton transport functions (Sbia et al., 1992). Calcium dependence of mediatophore assembly and function seems well supported (Israël et al., 1993; Morel and Israël, 2000; Malo and Israël, 2003; Dunant et al., 2009; Fujii et al., 2012). However, recent studies challenge the original c-ring fusion pore model about the role of V-ATPase in membrane fusion processes (Poea-Guyon et al., 2013; Bodzęta et al., 2017), suggesting a more complex scenario. Gap-junction proteins from mouse liver plasma membrane and the hepatopancreas of *Nephrops norvegicus* (arthropod) showed both high sequence and structural similarity to the above proteins, and their different relative orientation relative to the cytoplasm was observed (Finbow and Meagher, 1992; Finbow et al., 1993). In addition, the same proteolipid was found to be a constituent of both the ACh releasing mediatophore and the V-ATPase in *Torpedo* (Brochier and Morel, 1993), indicating more than one function of some of the related proteins. Based on microscopic and spectroscopic data we have provided evidence for a common structure for a class of membrane channels, that we named *Ductins*: The gap-junction-like structures isolated from *Nephrops norvegicus* were composed of a 16-kDa polypeptide, and the functional assembly was a star-shaped hexamer of the protein, of a four trans-membrane alpha helix (TMH) per monomer topology, arranged around a central channel (Holzenburg et al., 1993) [see left drawing in Figure 1 for an illustration, based on (Pali et al., 1995; Pali et al., 1997; Harrison et al., 1999; Pali et al., 1999)]. It was also shown that the *Nephrops* 16-kDa protein could substitute for the subunit c of V-ATPase in yeast yielding a functional hybrid enzyme (Holzenburg et al., 1993; Finbow et al., 1994). It had been long disputed but now it is established that subunit c (a *Ductin* protein) and other subunits of the V_o_ domain of V-ATPase play direct roles in some vesicle transport processes by facilitating membrane fusion, via intra- and inter-membrane subunit rearrangement and interaction with other fusion proteins, unrelated to the acidification role of V-ATPase (Galli et al., 1996; Shiff et al., 1996; Israël and Dunant, 1999; Di et al., 2010; El Far and Seagar, 2011; Strasser et al., 2011; Amendola et al., 2015; Couoh-Cardel et al., 2016; Rama et al., 2019).

### V-ATPase in autophagy

Intracellular and extracellular material destined for degradation are transported along the autophagy, endocytic, and phagocytic pathways, respectively. Their shared endpoint is the many lysosomes (in animal cells) and usually a single vacuole (in plants and fungi), where acidic hydrolases break down proteins, lipids, nucleic acids, and carbohydrates into building blocks for subsequent recycling and reuse in the cytosol. Lysosomal/vacuolar hydrolases function optimally in an acidic internal milieu (pH: 4.5-5.0), which is generated and maintained by the V-ATPase complex. Considering the fundamental, homeostatic role of autophagy and endocytosis both at the cellular and organismal levels, the most important function of V-ATPase is to promote these vesicle-mediated degradation pathways, but very little is known about the regulation of V-ATPase during these scenarios. Although the non-ubiquitously expressed V-ATPase isoform ATP6V0D2/subunit d2 was found to bind to Syntaxin 17, the autophagosomal SNARE that others and we have identified as a key competence factor enabling lysosomal fusion (Itakura et al., 2012; Takáts et al., 2013), this Syntaxin 17-binding ATP6V0D2/subunit d2 was found to be dispensable for lysosome acidification as it promotes autophagosome-lysosomal fusion (Xia et al., 2019). On the other hand since, at least in the above referred context, a physical and functional interaction of Syntaxin 17 and V-ATPase is firmly established and because ATPV0D is closely situated to the c-ring of the V_o_ subunit, interaction of the latter with Ca^2+^ may have a structural impact providing a mean for regulation. Unlike Mg^2+^, which supports both ATP hydrolysis and coupled vectorial proton transport of the purified holoenzyme, Ca^2+^ has been shown to facilitate only its ATPase activity. However, Ca^2+^ supports a coupled reaction having given that the holoenzyme is membrane embedded and there is a favourable membrane potential difference. There is no solid explanation for these findings, but Ca^2+^ binding to the holoenzyme most likely have a structural impact on the V-ATPase, and may serve thereby as a mean for quality control to support the coupled reaction only if the holoenzyme is properly positioned into a membrane, a regulatory role could not be exerted by Mg^2+^. This putative mechanism may act on some of the V_o_ subunits such as V_o_c (a *Ductin*, c-ring protein), as it is the part of the complex to interact with the internal side of the membrane and may be affected by membrane potential. We found that the V-ATPase complex itself is dispensable for autophagosome-lysosome fusion in *Drosophila* fat cells (even though its loss inhibits lysosome acidification), and interestingly, the vesicle fusion blocking effect of the commonly used V-ATPase inhibitor bafilomycin A1 could be attributed to Ca^2+^ dyshomeostasis within cells (Mauvezin et al., 2015). Thus, these data leave the question still open whether Me^2+^ (or Ca^2+^ in particular) have any direct effect on the V-ATPase in autophagy.

### F-ATPase and the mitochondrial permeability transition pore (mPTP)

The *Ductin* family also includes the c-ring protein subunit (c) of the ATP synthase (F-ATPase). A 16-kDa *Ductin* protein (such as, e.g., the 4TM V_o_c subunit) is basically a tandem repeat of the 8-kDa subunit c of F-ATPase (with 2 TMHs) based on sequence and structure similarity between subunit c of F-ATPase and the *Nephrops* and other 16-kDa *Ductin* proteins from different species (Holzenburg et al., 1993). V-ATPase works in the opposite sense as the better known F-ATP synthase, which normally synthesises ATP on the cost of trans-membrane delta pH (Borsch, 2013; Junge and Nelson, 2015; Nakanishi et al., 2019; Noji et al., 2020). F- and V-ATPases are true molecular motors, and the catalytic process (ATP hydrolysis or synthesis) and proton transport are strongly coupled via the rotary mechanism in both enzymes (Rawson et al., 2015; Sugawa et al., 2016; Yamato et al., 2016; Ferencz et al., 2017; Yanagisawa and Frasch, 2017; Kühlbrandt, 2019; Murphy et al., 2019; Kubo et al., 2020; Noji et al., 2020; Pinke et al., 2020; Frasch et al., 2022; Kishikawa et al., 2022). Mitochondria tightly regulate the permeability of their inner membrane to maintain efficient ATP synthesis. Stress events lead to dys-regulation of cellular [Ca^2+^], which causes loss of the inner membrane potential, and the process results in non-specific permeability transition pores, metabolic dysfunction and ultimately cell death (Carraro and Bernardi, 2016; Giorgio et al., 2018; Nesci, 2020). It is now established that if mitochondrial F-ATPase becomes a molecular target of Ca^2+^ it is one of the key events in the formation of the mitochondrial permeability transition pore (mPTP) (Nath, 2020; Algieri et al., 2021; Nesci, 2022a; Nesci, 2022b). The process of mPTP formation is complex and probably involves dissociation of F-ATPase dimers, then the F_1_-F_o_ domains, and it depends on many factors (Amodeo et al., 2017; Bonora et al., 2017; Mnatsakanyan and Jonas, 2020; Nath, 2020). Nevertheless, F-ATPase seems to be able to accommodate all the key factors (including Ca^2+^, Mg^2+^, adenine nucleotides, membrane potential, matrix pH, SH oxidants and reductants, etc.) that regulate mPTP activity (Giorgio et al., 2018). Several putative Ca^2+^ binding locations on the F-ATPase and related effects had been proposed (Giorgio et al., 2017; Mnatsakanyan and Jonas, 2020; Nath, 2020), but the [Ca^2+^]-dependent c-ring assembly appears to be a key step in the actual pore formation (Alavian et al., 2014; Halestrap, 2014; Bonora et al., 2017; Neginskaya et al., 2019; Mnatsakanyan and Jonas, 2020; Amodeo et al., 2021; Nesci, 2022a). (This c-ring pore assembly is somewhat similar to the gap-junctional arrangement of the V-ATPase V_o_ subunit c homologue of lobster hepatopancreas (Holzenburg et al., 1993; Pali et al., 1995).)

### Putative *Ductin* binding site(s) for divalent metal cations (Me^2+^)

*Ductins* can be defined as a family of homologous and structurally similar membrane proteins with 2 or 4 trans-membrane alpha-helices whose active forms are membranous ring- or star-shaped oligomeric assemblies and they provide various pore, channel, gap-junction functions, assist membrane fusion processes and also serve as the rotor c-ring domain of F- and V-ATPases. The multifunctional character puts the *Ductins* at the crossroads of a number of key biological processes (Dunlop et al., 1995; Finbow et al., 1995; Lautemann and Bohrmann, 2016). In a series of studies, we have characterised the 16-kDa lobster gap-junction protein as concern its membranous hexameric assembly, interaction with lipids and inhibitors, and the membrane location of some key residues (Jones et al., 1995; Pali et al., 1995; Pali et al., 1997; Pali et al., 1999; Pali et al., 2004). Most importantly, we have also shown that, as purified from the hepatopancreas of *Nephrops norvegicus*, the stable c-ring form of this *Ductin* protein contained a Me^2+^ binding site that was occupied by Cu^2+^, that could be removed by washing with EDTA. Titration with NiCl_2_ then yielded Ni^2+^ bound to the exchangeable Me^2+^ site (Pali et al., 2006). This site was found to be situated closer to Cys54 (of TMH2) and the C5 position of the lipid chains than to the C9-C14 fatty acid chain positions, possibly in the outer ring of TMHs. Back then we had not identified the function of this Me^2+^ binding site, but suggested that it might be involved in copper homeostasis. Further experiments with inhibitors and chelators (unpublished) suggested that Me^2+^ binding to the lobster protein might have a stabilising effect on the ring- or star-shaped assembly. It has been observed recently that excess Cu^2+^ caused the inhibition of vacuole fusion and V-ATPase function in yeast. In addition, a Cu^2+^-specific chelator rescued fusion, whereas a Cu^1+^-specific chelator had no effect on the inhibited fusion (Miner et al., 2019) (although the chelators used might not have penetrated inside the cells, which prevents excluding the possibility of the effect of intracellular Cu^1+^). This observation is in an apparent contradiction with earlier observations made on cucumber roots where both the hydrolytic and proton-transport activity of V-ATPase increased under copper stress (Kabała et al., 2013). Importantly, copper induced transcription of subunit c of V-ATPase was also observed, hinting that this subunit is a direct target of Cu^2+^ and that the effect of copper treatment on V-ATPase activity was actually the result of a compensatory mechanism provoked by copper toxicity in roots (Kabała et al., 2014). In addition, it is likely that the stabilising/destabilising [Me^2+^] effects are not unidirectional and uniform for all states of the enzyme and the c-ring (see below). For instance, in this case low effective [Cu^2+^] is probably sufficient for shifting the equilibrium towards assembled c-rings (ie., when V_o_ and V_1_ are dissociated) from monomeric c subunits, and low [Cu^2+^] is conditional to structural flexibility of the c-ring needed for assisting membrane fusion processes. On the other hand, high(er) [Cu^2+^] might over-stabilise the c-ring preventing its role in membrane fusion but promoting the formation of functional V_o_, but very high [Cu^2+^] might interfere with the intact enzyme possibly through different binding sites/mechanism (for instance by perturbing the V_o_-V_1_ association). These observations also further point toward a role of the V-ATPase in copper homeostasis under un-perturbed, none-stressed conditions (Eide et al., 1993; Pali et al., 2006; Schlecht et al., 2014). There have been further convincing arguments provided for Me^2+^ binding sites present in the c-ring (*Ductin*) assembly of the F- and V-ATPases, and it has also been proposed that different cations can bind (Pali et al., 2006) and even compete for binding sites on the F-ATPase under certain conditions (Van Walraven et al., 2002; Giorgio et al., 2017; Nath, 2020; Nesci, 2022a). Therefore it appears that the *Ductin* Me^2+^ binding sites are structurally flexible and not very specific.

### [Me^2+^]-dependent regulation of *Ductin* assembly and function

Systematic studies on the affinity profile of Me^2+^ binding to *Ductin* proteins are still missing but strongly needed for a better understanding of Me^2+^ effects on the function of these proteins. Nevertheless, it is clear from the above overview that all the various functions of the *Ductin* proteins have been documented to be sensitive to the presence of certain divalent cations, most frequently Cu^2+^ or Ca^2+^ ions, for most of the better known members of the family. Given that we found a prominent Me^2+^ binding site in a well-characterised *Ductin* protein, and in view of the great sequence and structure similarity within the family, we hypothesise that, in addition to the already known regulatory factors, Me^2+^ can structurally modulate the various functions of oligomeric *Ductin* assemblies via affecting their stability by reversible non-covalent binding to them (Figure 1). Other binding site(s) are still mostly un-identified and the detailed mechanism of Me^2+^ effects on *Ductin* assemblies are not yet known. However, it can be concluded that the sign of the—stabilising or destabilising—effect of Me^2+^ binding to *Ductin* assemblies depend on [Me^2+^] and on wether these assemblies are part of an intact rotary ATPase or their proton-conducting membrane domain, or the *Ductins* are free in a monomeric or oligomeric form in the membrane. For instance, the activity of F- and V-ATPases decrease at high [Me^2+^], probably because of interference with subunit-subunit interactions, and high [Me^2+^] might promote dissociation of the catalytic (F_1_, V_1_) from the transport (F_o_, V_o_) domains in these rotary enzymes. On the other hand, low [Me^2+^] is probably needed for assembling and stabilising the c ring from monomers. Indeed, a fine control of the stability of the assembly ranging from disassembled monomers through a loosely to tightly assembled c-ring is expected to render precise regulation of *Ductin* functions possible. For instance, in V-ATPase and F-ATPase loosening or tightening the c-ring of the rotor may decrease or increase, respectively, the efficiency of the catalytic function and the rate of the proton translocation. In order to posses a central pore, mediatophore and gap-junction *Ductin* proteins must be in the assembled c-ring form (Figure 1, left), which is promoted by binding of divalent cations. On the other hand, *Ductins* participating in membrane fusion processes need intra- and inter-membrane rearrangements and exchange of monomers, which assumes, at least temporarily, a loose c-ring form or monomeric *Ductins* (Figure 1, right), which in turn assumes low concentration of any structural Me^2+^, whereas high [Me^2+^] prevents rearrangement of the monomers. In addition, in the loose c-ring or monomeric state the lipid-protein interface is different from the sealed stable c-ring state (Figure 1), which renders different lipid-specific regulatory effects possible. It is very likely that there is an affinity profile of the binding site(s) for different divalent metal cations, but so far mostly Cu^2+^ and Ca^2+^ were reported to affect the various *Ductin* functions. It should be noted that a direct Me^2+^ binding effect on *Ductin* assemblies might be masked *in vivo* by other processes sensitive to the Me^2+^. Therefore reconstituted systems should be initially preferred for testing the above hypothesis and for further studies on the details of Me^2+^ binding to *Ductins* assemblies. Finally, since un-plugged c-ring pores in biomembranes are lethal to the host cells, expression systems aiming at purification of *Ductin* proteins should prefer low [Me^2+^], in order to prevent pore formation of the over-expressed monomers.
